# Cognitive function is associated with performance in time up and go test and with leptin blood levels in community-dwelling older women

**DOI:** 10.1038/s41598-024-60274-5

**Published:** 2024-04-29

**Authors:** Leonardo Augusto da Costa Teixeira, Luana Aparecida Soares, Liliana Pereira Lima, Nubia Carelli Pereira Avelar, Julia Araújo de Moura, Amanda Aparecida Oliveira Leopoldino, Pedro Henrique Scheidt Figueiredo, Adriana Netto Parentoni, Vanessa Amaral Mendonça, Ana Cristina Rodrigues Lacerda

**Affiliations:** 1 Programa de Pós-Graduação em Ciências da Saúde (PPGCS), Universidade Federal dos Vales do Jequitinhonha e Mucuri, Diamantina, MG Brazil; 2 Programa de Pós-Graduação em Ciências Fisiológicas (PPGCF), Universidade Federal dos Vales do Jequitinhonha e Mucuri, Diamantina, MG Brazil; 3 Programa de Pós-Graduação em Reabilitação e Desempenho Funcional (PPGReab), Universidade Federal dos Vales do Jequitinhonha e Mucuri, Diamantina, MG Brazil; 4 Departamento de Fisioterapia, Faculdade de Ciências Biológicas e da Saúde, Universidade Federal dos Vales do Jequitinhonha e Mucuri, Diamantina, MG Brazil; 5 Departamento de Fisioterapia, Universidade Federal de Santa Catarina, Araranguá, SC Brazil; 6 Programa de Pós-Graduação em Educação Física (PPGEF-UnB), Universidade de Brasília, Brasília, DF Brazil; 7 Faculdade de Ciências Médicas de Minas Gerais (FCMMG), Belo Horizonte, MG Brazil

**Keywords:** Immunology, Neuroscience, Biomarkers, Medical research

## Abstract

Considering the challenge that cognitive dysfunction and dementia represent to health is imperative to prioritize early diagnosis strategies and explore the pathophysiological mechanisms. There is no consensus on specific markers and physical tests that indicate cognitive decline in older. The objective of this study was to evaluate a panel of inflammatory biomarkers and physical function and investigate their association with cognitive function in community-dwelling older women. Seventy-one participants were included in this study. Cognitive function was assessed by Mini Mental State Examination, muscle strength using dynamometer, body composition using Dual X-ray absorptiometry, respiratory muscle strength using manuvacuometer, and physical function using the Short Physical Performance Battery and Time Up and Go (TUG) tests. Blood samples were collected to analyze a panel of inflammatory biomarkers. The cognitive function was associated with TUG (β = − 0.48; 95%IC = − 0.54 to − 0.21; *p* < 0.001), inspiratory muscle strength (β = 0.30; 95%IC = 0.005–0.03; *p* = 0.009), and leptin concentrations (β = 0.32; 95% IC = 0.001–0.006; 0.007). Time spent on TUG test and leptin levels accounted for 27% of variability in cognitive function independent of age. Poorer physical function with leptin plasma levels is associated with decreased cognitive function in older women. These findings contribute to comprehension of pathophysiology underlying cognitive decline and informing the development of new approaches to prevent, diagnose, monitoring and treat cognitive decline in aging.

## Introduction

Cognitive function is encompassing term used to refer to mental processes involved in acquiring knowledge, processing information and reasoning^1^. The most common domains that reflect cognitive function are learning, attention, memory, perception, language, and decision making^1,2^. Cognitive impairment in midlife is associated with a higher risk of dementia later in life^3^ and is a common characteristic of dementias such as Alzheimer's and Parkinson's, which share similar pathological and inflammatory pathways^4^. In the older people, cognitive disfunction is highly prevalent around the world and is associated with loss of independence and the ability to effectively perform daily activities^4,5^.

Neuroinflammation plays a crucial role in the development of dementia^6,7^. Evidence has demonstrated that individuals with cognitive impairment presents an inflammatory imbalance, characterized by higher systemic levels of pro-inflammatory markers compared to healthy individuals^6,8,9^. In this sense, a series of studies aimed to establish potential mechanisms underlying cognitive impairment and identify biological markers^9^. The adipokines are hormones secreted by adipose tissue involved in energy homeostasis and metabolism regulation and have already been reported as a contributing factor to the increased risk of dementia in older adults^10–12^.

Tumor necrosis factor (TNF), interleukin-1β (IL-1β), and C-reactive protein have been associated with an elevated risk of dementia^13^, while interleukin-2 (IL-2), IL-6, and IL-13 have been identified as important biomarkers related to cognitive decline^8^. Changes in the blood level of brain-derived neurotrophic factor (BDNF) appear to occur early in the development and progression of cognitive impairment^14,15^, and lower leptin levels have been observed in individuals with dementia compared to those without^12,16^, suggesting a possible involvement of both inflammatory mediators in the cognitive function of older people^7,11^.

Although previous studies have demonstrated associations between muscle mass, muscle strength and physical function tests with cognitive function, demonstrating that better physical capacity is associated with better cognition^5,17^, there are gaps in the literature regarding their relationship with inflammatory biomarkers^16,18,19^. Studies investigating a possible diagnostic using biomarkers and that may point to a probable inflammatory pathway related to cognitive decline are necessary, as they may contribute to the development of clinical or laboratory screening strategies for identifying cognitive dysfunction in older adults, as well pointing the potential treatment strategies^8,9,19,20^.

Therefore, the objective of the current study was to assess the cognitive function of community-dwelling older women and examine its association with physical assessment tests and a panel of inflammatory biomarkers. Additionally, the study aims to offer insights into strategies for monitoring cognitive decline and facilitating early diagnosis of cognitive dysfunction.

## Methods

### Design

This is an exploratory, cross-sectional study that received approval from the Ethics Committee of the Universidade Federal dos Vales do Jequitinhonha e Mucuri (UFVJM) under protocol number 1.461.306. The study was conducted according to the guidelines of the Declaration of Helsinki and all participants provided informed consent by signing a consent form. The assessments were conducted at the Laboratório de Fisiologia do Exercício (LAFIEX) and Laboratório de Inflamação e Metabolismo (LIM) at UFVJM, between June 2016 and June 2017.

### Sample

The study recruited community-dwelling older women residing in Diamantina, Minas Gerais, Brazil, based on their registration at the Basic Health Units (BHU) of the primary health care centers. Home visits were conducted for all participants, during which they completed a questionnaire providing information about clinical conditions. The inclusion criteria for participation were as follows: a woman, aged over 65 years, functionally independent in the community, and capable of performing the subsequent assessment procedures.

Exclusion criteria were applied to individuals who demonstrated cognitive impairment based on their education level, as determined by scores on the Mini-Mental State Examination (MMSE). Other exclusion criteria included individuals with neurological sequelae, recent hospitalization within the past three months, fractures in the upper or lower limbs within the past six months, acute musculoskeletal disorders that could interfere with the proposed physical assessments, acute respiratory or cardiovascular diseases that would prevent the performance of respiratory measurements maneuvers, acute-phase inflammatory diseases, active neoplasms within the last five years, individuals under palliative care, and those using anti-inflammatory medications or drugs that affect the immune system.

### Procedures

The evaluations were conducted over a period of three separate days. On the first day, a home visit was made where eligible participants signed the written informed consent, underwent clinical health interviews, and had their cognitive function assessed.

On the second day, participants were conducted to the laboratory at 8:00 a.m. after fasting from food, beverages, and medications. They underwent assessments of body composition and physical function tests. Following a 15-min break for a standardized snack and rest, all participants performed the physical performance tests.

On the third visit, which took place 24 h after the body composition and physical tests, blood samples were collected from the participants for the analysis of inflammatory biomarkers. Sampling information and procedures is also available in elsewhere^21,22^.

### Assessment of cognitive function

Cognitive function was assessed using the MMSE, which was chosen as a sample eligibility criterion due to its widespread use in tracking cognitive impairment^19^. This assessment tool includes questions that evaluate five dimensions: concentration, language/praxis, orientation, memory, and attention. The MMSE has a maximum score of 30 points, and the cutoff points are adjusted based on an individual's level of education^2^.

### Assessment of anthropometric values and body composition

The volunteers' body mass was measured using a digital scale (Welmy, model 110) with a precision of 0.1 kg and a resolution of 0.1 kg. Height was assessed using a stadiometer attached to the scale, with a precision of 0.5 cm. The participants were instructed to remove their shoes and wear light clothing during the measurements. Body mass index (BMI) was calculated by dividing the body mass (in kilograms) by the square of the height (in meters)^23^.

For the evaluation of body composition, Dual X-ray Absorptiometry (DXA) was used. Specifically, a Lunar Type DPX machine with Encore software version 2005 was utilized. This assessment method provided information on variables such as body fat, lean muscle mass, and bone mineral density. All body composition evaluations were conducted by the same assessor to ensure consistency and minimize inter-rater variability.

### Assessment of muscle strength

Handgrip strength (HGS) was assessed using a Jamar dynamometer®. Participants were instructed to sit in a comfortable position with their hand in a neutral position, elbow flexed, and shoulder in a neutral position. The participant then performed an isometric contraction by squeezing the handles of the dynamometer with their dominant hand. The measurement of HGS was recorded in kilogram-force (kgf). To ensure accuracy and reliability, three measurements were taken, and the average of these measurements was used for the analysis^24^.

### Assessment of respiratory muscle strength

Respiratory muscle strength was assessed by measuring the maximum inspiratory pressure (MIP) and the maximum expiratory pressure (MEP) using a manuvacuometer (model MV-150/300, Ger-Ar Comércio e Equipamentos Ltda.®). The participants were seated, with their feet flat on the floor, without support for the upper limbs, and using a nose clip. MIP was measured from the residual volume and MEP from total lung capacity. The maneuvers were repeated a maximum of five times, considering three acceptable maneuvers and maximum sustained respiratory efforts for at least two seconds. Acceptable measurements were those without air leaks and that obtained a variation of ≤ 10% of the maximum value found. Between each measurement, a minimum interval of one minute was established for the volunteer to recover^25^.

### Physical function tests

The participants underwent two assessments to evaluate their physical function including the Short Physical Performance Battery (SPPB) and the Time Up and Go (TUG) test.

The SPPB is a battery of tests used to assess lower limb function in older adults. It consists of three tests: static body balance, lower limb muscle strength, and gait speed. Each test is scored on a scale ranging from zero to 4 points, with a maximum total score of 12 points. A higher score indicates better performance in physical function^26^.

The TUG test involves documenting the time taken by an individual to rise from a chair, walk three meters, turn around an obstacle, return to the chair, and sit down again. The test measures functional mobility and a longer time to complete the test indicates poorer functional performance^27^.

### Analysis of blood inflammatory biomarker

Blood samples were collected at 8 a.m. from the antecubital fossa of the upper limb using disposable materials. Participants were required to fast for 10 h, refraining from consuming food, beverages, and medications during this period. The collection was performed using vacutainer bottles containing heparin in a sterile environment. Immediately after collection, the blood samples were centrifuged at 3000 rpm for 10 min. Following centrifugation, plasma samples were carefully extracted and stored at − 80 °C for a duration of six months before analyzed.

The levels of adiponectin, brain derived neurotrophic factor (BDNF), interleukin (IL)-2, IL-4, IL-5, IL-10, tumoral necrosis factor (TNF), leptin, resistin, and soluble receptors of TNF (sTNFr)-1 and sTNFr-2 were analyzed using the enzyme-linked immunosorbent technique (ELISA) with the Duo-Set kit from R&D Systems, Minneapolis, USA. The plasma levels of interferon (IFN), IL-6 and IL-8 were measured using a cytometric bead array kit from BD Bioscience, San Jose, CA, according to the manufacturer's protocol. The samples were acquired using a FACSCanto flow cytometer from BD Bioscience and analyzed using the FCAP array v1.0.1 software from Soft Flow^28^.

### Statistical analyses

The statistical analyses were conducted using IBM SPSS Statistics version 22.0 (Armonk, NY, USA) and Med-Calc Statistical Software version 13.1 (Ostend, Belgium). The normality of the data was assessed using the Kolmogorov–Smirnov test. Continuous variables were presented as mean and standard deviation (for normally distributed data) or median and interquartile range (for non-normally distributed data), depending on the distribution of the data. Group comparisons were performed using the ANOVA test and independent t-test, and statistical significance was determined when the *p*-value was less than 0.05.

The classification of groups based on cognitive function was determined using percentiles. Descriptive frequencies of MMSE scores were analyzed, and the 25th, 50th, and 75th percentiles were observed to establish cutoff points for the three groups. Three cognitive function groups were determined: values below 21 indicated the lowest cognitive function, values between 21 and 24 indicated moderate cognitive function, and values above 24 indicated higher cognitive function. The Kruskal Wallis one way test was used for the group comparisons and statistical significance was identified with *p* value less than 0.05.

Spearman correlation analysis was conducted to examine the relationships between cognitive function, body composition variables, physical performance, and inflammatory biomarkers. Variables that showed a correlation with the MMSE score (*p* were included in the regression analyses. Univariate and stepwise multivariate linear regression analyses were performed to determine the factors influencing cognitive function. The linear regression analysis followed four assumptions: linearity, residual distribution, homoscedasticity, and absence of multicollinearity. Scatter plots were used to assess the linearity between independent variables and residuals, while a histogram was used to examine the distribution of residuals. Homoscedasticity was confirmed when the residuals were evenly distributed along the regression line. The absence of multicollinearity was determined by checking the variance inflation factor (VIF) values, with values below 10.0 indicating no multicollinearity. The presence of autocorrelation in the data was assessed using the Durbin–Watson test. Statistical significance was set at 5%.

### Institutional review board statement

The study will be conducted according to the guidelines of the Declaration of Helsinki and was approved by the Institutional Ethics and Research Committee of Federal University of Jequitinhonha and Mucuri Valleys (nº protocol 1.461.306 on 22 March of 2016).

## Results

**Figure 1:**
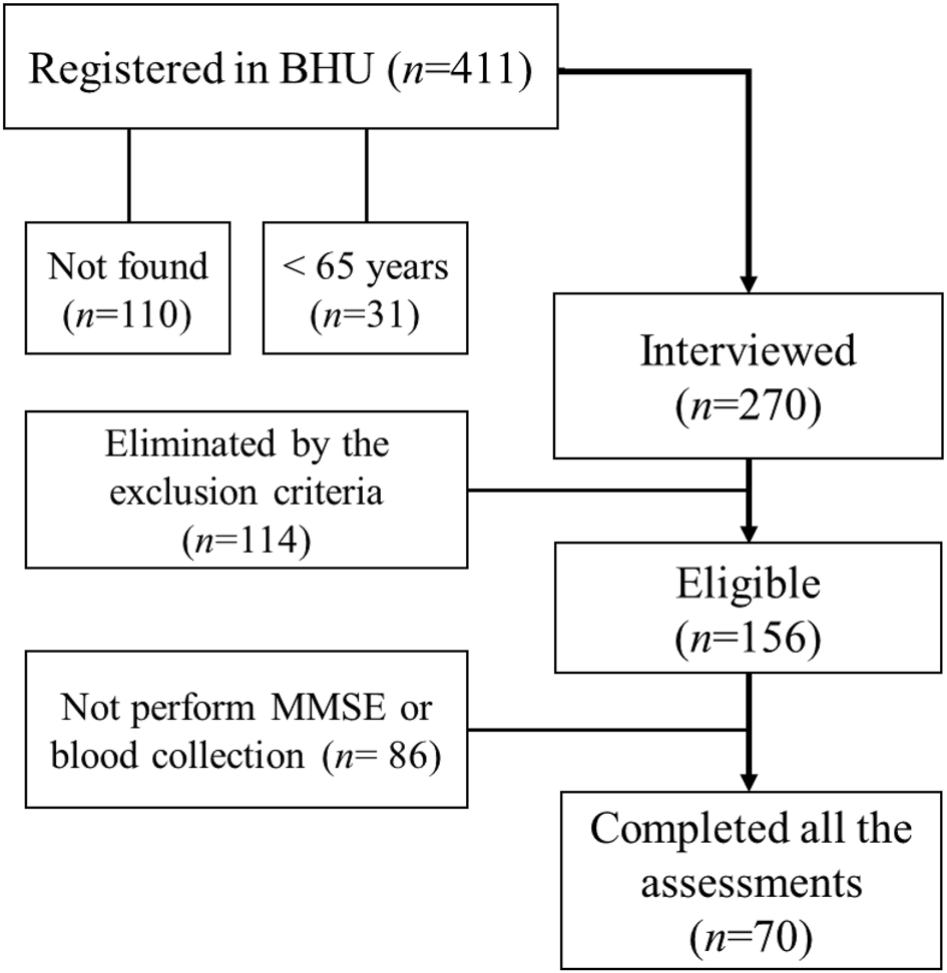
Flowchart of the sample recruitment.

This is a secondary analysis of a previous study on sarcopenia and biomarkers^21^. A total of 411 older women were initially identified based on their registration at the BHU. However, 73 addresses could not be located, and 31 individuals were found to be below the age requirement of 65 years. Subsequently, 270 older women were interviewed in their homes, but 120 of them did not meet the inclusion and exclusion criteria. As a result, 156 community-dwelling older women remained eligible for the evaluation procedures. One individual did not provide MMSE values and eighty-five did not undergo blood sampling. Finally, 70 older women successfully completed all assessments and were included in the present study (Fig. 1).

The characteristics of the participants were mean age of 75 years (± 7); weight of 59 kg (± 11); height of 1.50 m (± 0.05); BMI of 26 kg/m^2^ (± 4); handgrip strength of 19.8 kgf (± 6.4); muscle mass index of 6.38 kg/m^2^ (± 1.06); SPPB score of 8.6 points (± 2); TUG score of 11.6 s (± 4); MIP of 72.4 cmH_2_0 (± 45.1); MEP of 63.9 cmH_2_0 (± 34.9). Of note, 25.7% of the older women had the lowest cognitive function, 25.7% had an average cognitive function, and 48.6% had the highest cognitive function. Comparisons between groups regarding physical performance variables and the panel of biomarkers are shown in Table 1. (Table 1).

**Table 1:** Anthropometric characteristics, body composition, physical function, and inflammatory biomarkers of older women in the community (*n* = 70).

Variables	Cognitive function			*p* value
	Lower (n = 18)	Average (n = 18)	Higher (n = 34)	
Age (years)	76.5 (8.54)	77.5 (8.01)	73.03 (6.01)	0.09
BMI (kg/m^2^)	24.81 (4.14)	25.63 (5.25)	27.07 (4.15)	0.14
HGS (kgf)	18.12 (6.30)	19.99 (6.50)	20.69 (6.41)	0.19
SMI (kg/m^2^)	6.11 (1.07)	6.41 (1.16)	6.5 (1)	0.59
SPPB (score)	7.89 (2.44)	8.28 (2.19)	9.18 (1.60)	0.12
TUG (time)	13.93 (6.26)	12.26 (2.59)	9.98 (1.99)	0.002*^bc^
MIP (cmH_2_0)	60.83 (38.24)	55.56 (27.80)	87.94 (51.31)	0.03*^bc^
MEP (cmH_2_0)	53.61 (37.33)	56.67 (25.02)	73.24 (36.51)	0.20
Biomarkers				
Adiponectin (ɳg/mL)	49.38 (4.72)	48.41 (8.54)	49.83 (72.40)	0.89
BDNF (ɳg/mL)	27.73 (12.24)	22.22 (5.86)	25.28 (9.16)	0.47
IFN (pg/mL)	1.31 (0.23)	1.43 (0.22)	1.76 (2.18)	0.47
IL-2 (pg/mL)	4.04 (0.29)	4.23 (0.37)	6.22 (12.30)	0.24
IL-4 (pg/mL)	1.99 (0.16)	2.05 (0.25)	2.79 (4.48)	0.68
IL-5 (pg/mL)	0.86 (0.35)	1.48 (1.88)	0.95 (1.05)	0.14
IL-6 (pg/mL)	16.86 (3.17)	17.67 (5.56)	17.72 (4.05)	0.73
IL-8 (pg/mL)	26.21 (11.9)	22.03 (5.01)	23.41 (8.99)	0.33
IL-10 (pg/mL)	1.54 (0.21)	1.82 (1.09)	2.63 (4.84)	0.54
Resistin (ɳg/mL)	16.06 (4.31)	16.25 (3.24)	16.46 (3.41)	0.92
sTNFr-1 (ɳg/mL)	45.36 (38.17)	34.12 (30.19)	37.71 (31.47)	0.44
sTNFr-2 (ɳg/mL)	21.97 (7.17)	21.76 (3.76)	20.55 (3.92)	0.49
TNF (pg/mL)	1.07 (0.13)	1.07 (0.17)	1.57 (2.70)	0.54

BMI, body mass index; HGS, handgrip strength; SMI, skeletal muscle index; SPPB, Short Physical Performance Battery; TUG, time up and go; MIP, maximal inspiratory pressure; MEP, maximal expiratory pressure; BDNF, brain derived neurotrophic factor; IFN, interferon; IL, interleukin; TNF, tumoral necrosis factor; sTNFr, soluble receptor of TNF. ^b^statistical differences between groups 1 and 3; ^c^statistical differences between groups 2 and 3; *p < 0.05.

**Figure 2:**
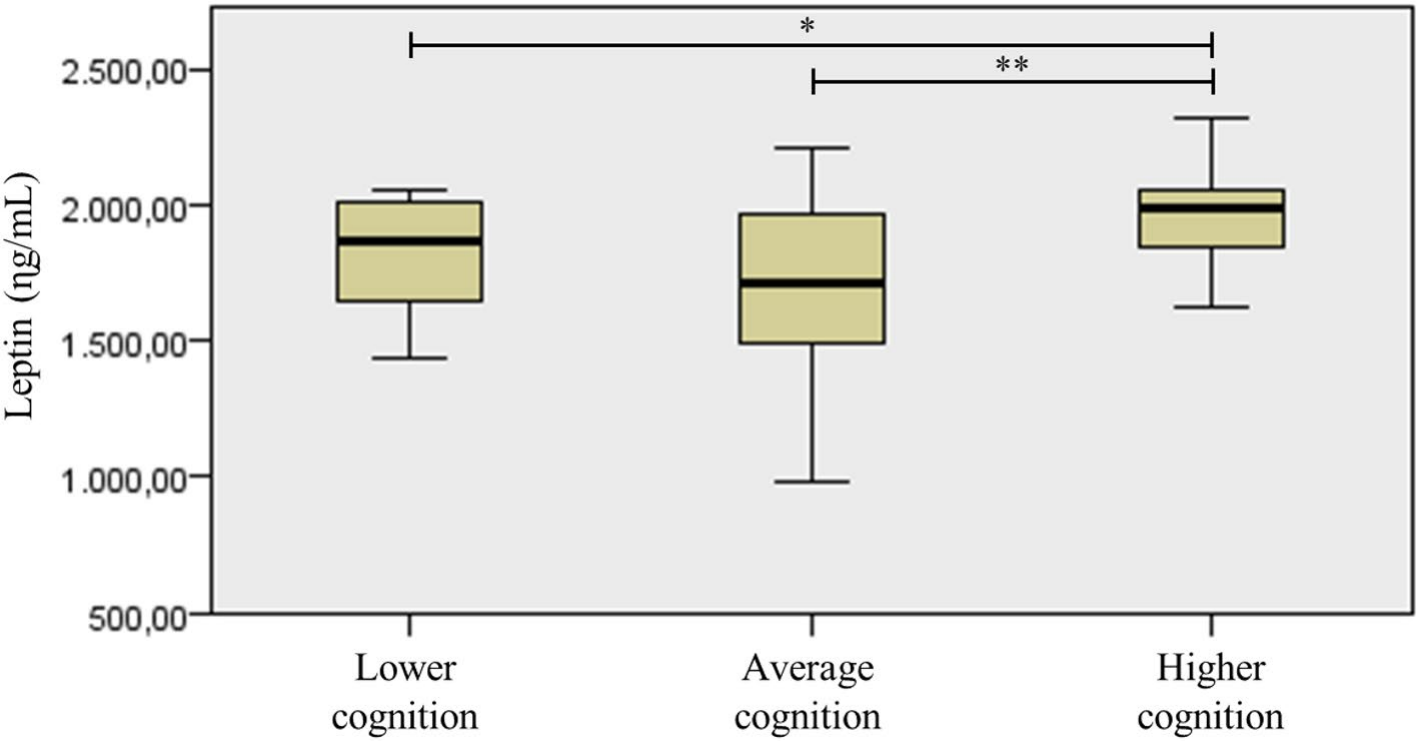
Distribution of leptin blood levels between cognitive function groups in community-dwelling older women. (n = 70). Kruskal Walis test for independent samples was used for comparison between groups stratified by cognitive function and their respective plasma leptin blood levels. Differences with *p < 0.05 **p > 0.01.

Analyzing the distribution of the biomarker panel, differences between groups were found in plasma leptin blood levels, the groups with low and medium cognitive function differed significantly from the group with higher cognitive function scores (Fig. 2).

Cognitive function in older women in the community was found to have negative correlations with the time spent on the TUG test and positive correlations with BMI, respiratory muscle strength, and plasma levels of leptin. The Spearman's correlation analysis showed a significant correlation between cognitive function and SPPB score (r = 0.24, *p* < 0.04), time on the TUG test (r = − 0.48, *p* < 0.001), MIP (r = 0.29, *p* = 0.01), IL-6 (r = 0.24, *p* = 0.04), and Leptin (r = 0.37, *p* = 0.001) in community-dwelling older women.

In the univariate regression analysis, cognitive function was the dependent variable and showed associations with SPPB score, inspiratory muscle strength and time on the TUG test (Table 2).

**Table 2:** Independent contributors to cognitive function in community-dwelling older woman (*n* = 70).

Independent variables	Univariate			Multivariate				
	β	R^2^ adjusted	95% IC	*p*	R^2^	R^2^ adjusted	DW	*p*
SPPB (score)	0.28	0.07	0.07–0.80	0.01*	–	–	–	NS
MIP (cmH_2_0)	0.30	0.08	0.005–0.03	0.009*	–	–	–	NS
TUG (time spent)	− 0.48	0.22	− 0.54 to − 0.21	< 0.001*	0.29	0.27	1.63	0.01*
Leptin (ɳg/mL)	0.32	0.09	0.001–0.006	0.007*				

DW, Durbin Watson; IC, confidence interval; BMI, body mass index; HGS, handgrip strength; SMI, skeletal muscle index; SPPB, Short Physical Performance Battery; TUG, time up and go; MIP, maximal inspiratory pressure; MEP, maximal expiratory pressure; IL-6, interleukin-6; β, beta coefficient; R^2^ adjusted: adjusted coefficient of determination. NS, Non-significance. Cognitive function as a dependent variable. *p-value < 0.05.

**Figure 3:**
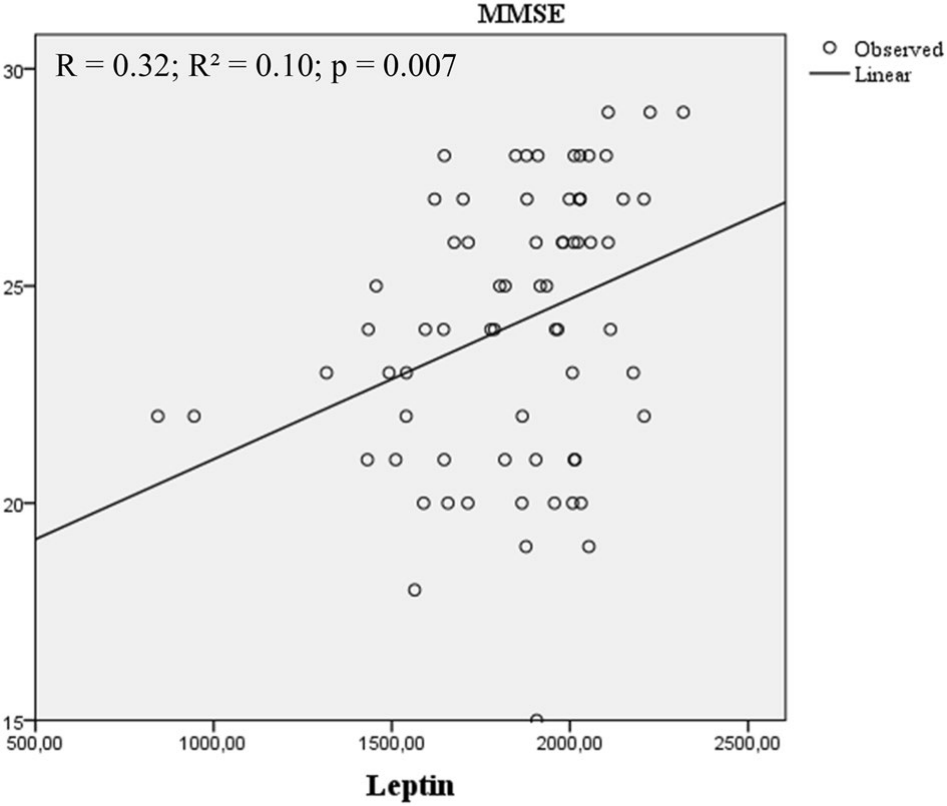
Association between cognitive function and leptin blood levels in community-dwelling older woman (n = 70). Spearman correlation revealed higher cognitive function values are correlated and associated with higher levels of leptin blood levels. *Abbreviation* MMSE, Mini Mental State Examination score; Leptin = ɳg/mL.

Analyzing the panel of biomarkers, cognitive function was associated with leptin blood levels (Fig. 3).

In the multivariate analysis, including physical function variables, inspiratory muscle strength, and biomarkers as independent variables, the TUG test and Leptin were the best predictors of cognitive function (Table 2). Additionally, when the data were adjusted for age, the results remained the same. In clinical terms, leptin concentrations were associated and explained 9% of the variability in cognitive function in community-dwelling older women. Worse performance on the TUG test also associated to cognitive function explained 22% of the variability in the scores of the MMSE. Lastly, the combination to time spent in TUG test and Leptin concentrations formed the best predictors to explain the variability in cognitive function of community-dwelling older women (Table 2).

## Discussion

In general, associations were found between cognitive function with physical and inflammatory aspects in community-dwelling older women. In this sample, the lower cognitive function was not significantly comparing groups related to age, BMI, or handgrip strength. The results indicate that cognitive function is associated with worse physical performance, including measures of gait and balance (TUG test), inspiratory muscle strength, and using a panel of inflammatory biomarkers, plasma levels of leptin. In addition, the potential predictors of cognitive function were time spent on the TUG test and plasma leptin blood levels, explaining 22% and 9% respectively, of the change in the cognitive function.

As already demonstrated in the literature, the TUG test was inversely associated with cognitive function, and older women with lower MMSE scores presented worse performance on the test. It is not yet clear whether the cognitive function directly affects physical performance, and it is speculated that older people with low cognitive function may have less understanding, processing of information and execution of the test, which demands attention, balance, agility and gait speed^27,29^. Similarly, impaired respiratory muscle function was also associated with lower cognitive function, suggesting a potential link between respiratory muscle strength and cognition in older adults^30^. These findings hold potential clinical utility in facilitating the screening and identification of cognitive decline in older women, utilizing simple physical tests that can be administered even before the onset of dementia.

The World Health Organization (WHO) published the Integrated Care for Older People (ICOPE) framework to guide the assessment and promotion of the intrinsic capacity of older people, which refers to the combination of physical and mental capabilities^31^. The domains assessed are cognition, mobility, psychological functions, vitality and sensory^32^. Evidence suggests that impairment of intrinsic capacity is prevalent in community-dwelling older people, particularly in locomotor and cognitive capacity^31,32^. Additionally, biomarkers are proposed as alternative to evaluate the inflammatory condition and intrinsic capacity of older people^33^. In our analyses, the multivariate regression analysis revealed that time spent on TUG test and leptin blood levels together accounted for a significant proportion (approximately 27%) of the variability in MMSE scores. Although the degree of association is low, it is worth highlighting that the results were significant and independent of age. Therefore, these findings indicate that both factors may have significant roles in assessing cognitive function and suggest a promising biomarker that could be utilized for monitoring the cognitive decline in older women.

Leptin is a hormone derived from adipose tissue, its receptors are widely expressed in many extrahypothalamic brain regions, including the hippocampus, brainstem, and cerebellum^10,11,34^. It is well documented that leptin signals information about the state of fat stores to the hypothalamic nuclei, which in turn control eating behavior, body weight, and endocrine control of energy balance^34,35^. This hormone appears to play a role in the synaptic plasticity of hippocampal neurons, and in the long-term neuronal potentiation that are crucial for learning and memory^7,34,36,37^. In animal model (C57BL/6 male mice) fed a standard diet, the presence of leptin was able to modulate neurotransmission in the SC-CA1 pathway, while a hypercaloric regimen resulted in hippocampal resistance to the functional effects of leptin^37^. Suggesting that obesity associated with possible leptin resistance may be associated with increased risk of dementia^37^. Additionally, both leptin depletion and resistance may contribute to the neural plasticity deficits typical of diseases such as Alzheimer's^11,37^.

Consistent with our findings, higher leptin levels in older adults have been shown to be related to less cognitive decline, regardless of comorbidities and body fat levels^7,38^. These findings suggest a negative correlation between leptin levels and the risk of Alzheimer's disease, further supporting the hypotheses that low leptin levels are associated with the risk of dementia in adults and the older^7,39,40^. One of the main risk factors for developing Alzheimer's disease is aging^41^. There appears to be a hormonal decline that results in leptin sensitivity with age, and related mechanisms is still not entirely clear^7,38,39^. Leptin uptake by hypothalamic nuclei also decreases with age, and this correlates with reduced expression levels of leptin receptors^41^. Furthermore, in older, leptin levels were positively correlated with cerebral structure, again suggesting a protective effect against age-related atrophy^7,42^.

Amyloid-β accumulation is a key event mediating cognitive deficits in Alzheimer's disease, as this protein promotes synaptic dysfunction and triggers neuronal death^41^. Evidence suggests that leptin levels are markedly attenuated in Alzheimer's patients. Leptin is also a potential cognitive enhancer as it facilitates cellular events underlying hippocampal learning and memory. This is due to the action of leptin that counteracts multiple harmful events observed in the pathogenesis of Alzheimer's disease, from aberrations in hippocampal synaptic function to changes in the expression of proteins related to the disease and prevention of neuronal death. Our findings reinforce the emerging consensus that the leptin system is a promising biomarker for tracking or monitoring cognitive decline and potential therapeutic target in Alzheimer’s disease and cognitive decline. Furthermore, supporting the hypothesis of a potential protective effect on cognitive decline in community-dwelling older women^34,40,42^. In addition, some studies have been proving the beneficial therapeutic action of the presence of leptin positively modulating brain recovery, hippocampal synaptic function, and maturation processes^7,34,37,41,42^.

Additionally, our findings may point to a potential role of inflammation in cognitive decline^13^. Although not statistically significant, higher levels of the BDNF, IL-8 and soluble receptor sTNFr-1 were observed in older women with lower cognitive function. The BDNF has been proposed as a biomarker for impaired memory and cognitive function in older women^14,15^ and elevated levels of IL-8 and sTNFr-1 have been linked to declining intrinsic capacity or sarcopenia in the older^21,43^.

It is noteworthy our study has limitations including the cross-sectional design, which limits causal inference. The exclusion criteria were strict, and individuals with confirmed cognitive impairment were not participated in the evaluations. Additionally, considering that inflammation in aging is different between men and women^44^, our sample consisted only of older women from the community, which may limit generalizability to other populations. Another limitation of the present study is the fact that it did not explore the participants' diet, as it has been proven that a high-fat diet can interfere with blood leptin concentrations^37^, compared to a standard diet. Advances in research that evaluates a greater number of participants, including men, and investigating in depth the dietary profile of the participants, and that can stipulate cutoff points for detecting using inflammatory biomarkers of cognitive impairment in the older are necessary.

Finally, our findings suggest that TUG test performance, inspiratory respiratory muscle strength, and lower blood levels of leptin may indicate cognitive decline in community-dwelling older women. We provide valuable information that has clinical and research implications that aim to identify cognitive dysfunction and Alzheimer's disease early or pursue therapeutic strategies. Further studies are needed to explore the underlying mechanisms and validate this potential biomarker for cognitive impairment screening.

## Data Availability

The data presented in this study are available on request from the corresponding author. The data are not publicly available due to the privacy guarantee of the data collected individually.
